# Comprehensive genomic profiling: Does timing matter?

**DOI:** 10.3389/fonc.2023.1025367

**Published:** 2023-02-14

**Authors:** Bicky Thapa, Gulrayz Ahmed, Aniko Szabo, Mandana Kamgar, Deepak Kilari, Maahum Mehdi, Smitha Menon, Sherin Daniel, Jonathan Thompson, James Thomas, Ben George

**Affiliations:** ^1^ Division of Hematology and Oncology, Medical College of Wisconsin, Milwaukee, WI, United States; ^2^ Department of Biostatistics, Medical College of Wisconsin, Milwaukee, WI, United States; ^3^ Medical School, Medical College of Wisconsin, Milwaukee, WI, United States

**Keywords:** comprehensive genomic profiling, metastatic solid tumors, disparities, best practice, precision oncology

## Abstract

**Purpose:**

There is variability in utilization of Comprehensive Genomic Profiling (CGP) in most of the metastatic solid tumors (MST). We evaluated the CGP utilization patterns and its impact on outcomes at an academic tertiary center.

**Patients and Methods:**

Institutional database was reviewed for CGP data in adult patients with MST between 01/2012 – 04/2020. Patients were categorized based on interval between CGP and metastatic diagnosis; 3 tertiles of distribution (T1-earliest to the diagnosis, T3-furthest), and pre-mets (CGP performed prior to diagnosis of metastasis). Overall survival (OS) was estimated from the time of metastatic diagnosis with left truncation at the time of CGP. Cox regression model was used to estimate the impact of timing of CGP on survival.

**Results:**

Among 1,358 patients, 710 were female, 1,109 Caucasian, 186 Afro-Americans, and 36 Hispanic. The common histologies were lung cancer (254; 19%), colorectal cancer (203; 15%), gynecologic cancers (121; 8.9%), and pancreatic cancer (106; 7.8%). Time interval between diagnosis of metastatic disease and CGP was not statistically significantly different based on sex, race and ethnicity after adjusting for histologic diagnoses with 2 exceptions - Hispanics with lung cancer had delayed CGP compared to non-Hispanics (p =0.019) and females with pancreas cancer had delayed CGP compared to males (p =0.025). Lung cancer, gastro-esophageal cancer and gynecologic malignancies had better survival if they had CGP performed during the first tertile after metastatic diagnosis.

**Conclusion:**

CGP utilization across cancer types was equitable irrespective of sex, race and ethnicity. Early CGP after metastatic diagnosis might have effect on treatment delivery and clinical outcomes in cancer type with more actionable targets.

## Introduction

The variable natural history as well as disparate treatment responses noted even among tumors of similar histologic origin have largely been attributed to inherent differences in tumor biology and/or host response. Attempts at elucidating differences in tumor biology that can be parlayed into therapeutic options have led to the identification of several predictive and/or prognostic biomarkers across multiple cancer types, emphasizing the relevance of precision medicine in cancer treatment. The rapidly expanding horizon of precision therapeutics and a clear recognition that the complex biology of tumors cannot be adequately characterized by small panels of biomarkers. This led to the development of comprehensive biomarker panels and utilization of high throughput technology to enable better turnaround time (TAT) as well as cost effectiveness. Comprehensive Genomic Profiling (CGP) is now widely utilized in clinical practice -both academic and community settings- based on its predictive and diagnostic capabilities (1). Furthermore, tumor testing with CGP panels have facilitated patient enrollment in pivotal, biomarker enriched clinical trials paving the way for several FDA approved targeted therapies (2, 3).

Despite the widespread utilization of CGP in clinical practice, there is lack of standardization as it pertains to utilization of such panels. First, there are several commercial CGP panels available with varying levels of regulatory authorization and insurance reimbursement in addition to numerous ‘home grown’ institutional panels. Second, the timing of CGP in relation to a diagnosis of cancer varies widely based on awareness and practice patterns of oncologists. While most clinicians employ CGP for patients with metastatic solid tumors, there is no consensus on the optimal timing to perform CGP – is it better to perform the test soon after a diagnosis of metastatic disease or when standard treatments are no longer effective? Third, there is a lack of uniformity in CGP panels utilized by oncologists in the same practice and even within the same disease histology, making clinically annotated, biomarker-based data mining strategies cumbersome. Fourth, there is limited data on patient access to CGP based on racial, ethnic, and social disparities, in the absence of clear guidelines for such testing (4–7).

Recognizing the existing gaps in enterprise-wide streamlined utilization of CGP, we reviewed CGP data on all patients with solid tumors at our institution to understand test utilization patterns and its impact on clinical outcomes. Further, we propose an algorithm that can serve as an institutional framework for optimal CGP selection and utilization.

## Patients and methods

After IRB approval, this retrospective study was conducted in patients at our academic cancer center who underwent CGP from Jan 2012 to April 2020 at our institution. Patients diagnosed with metastatic solid tumors (MST), age>18 years, and adequate follow up were included in the study. All MST patients with CGP performed utilizing the Foundation One assay were identified through the physician-facing portal of Foundation Medicine and the institutional clinical data warehouse. We limited our analysis to patients who had CGP performed utilizing the Foundation one assay since that is the most commonly utilized assay at our institution. Other somatic CGP assays comprised approximately 10% of tests performed at our institution during this period and hence was not included in this analysis. Patient demographics and clinical information were collected by interrogating the electronic medical record. All patients with MST who underwent CGP at our institution were divided into diagnostic categories broadly based on the primary organ system involved (primary origin of malignancy) rather than the specific histologic diagnosis of primary malignant disease (**Supplementary table 1**).

Patients who had CGP performed were divided into four groups within each diagnostic category based on timing of the test in relation to their metastatic diagnosis; three tertiles of distribution (T1- closest to the diagnosis, T3-furthest) and pre-mets (patients who had CGP performed prior to metastatic diagnosis).

### Statistical analysis

Descriptive statistics were calculated for the study. Quantile regression was used to model median time of CGP relative to the time of metastatic diagnosis, including obtaining confidence intervals and multivariable regression modeling. Kruskal-Wallis test was used for between-group comparisons. Overall survival (OS) was estimated using the Kaplan-Meier method from the time of metastatic diagnosis with left truncation at the CGP time. Additionally, Kaplan-Meier conditional survival probabilities were computed for each conditioning time based on CGP test times (diagnosis, 1^st^, and 2^nd^ tertiles of the CGP time within diagnostic category). Cox regression model was used to estimate the impact of the time of CGP grouped by tertiles on survival for individual cancer types, with stratified by diagnostic category for the pooled data.

Survival was calculated from the time of metastatic diagnosis, rather than the time at which CGP was performed. Additionally, the follow-up was left-truncated at the time of the CGP to account for the “immortal” time before the CGP test. These choices ensure that the hazard of death is only compared between subjects who are alive the same time after diagnosis, after a CGP test, but with the test potentially performed earlier or later in the disease course.

## Results

**Figure 1:**
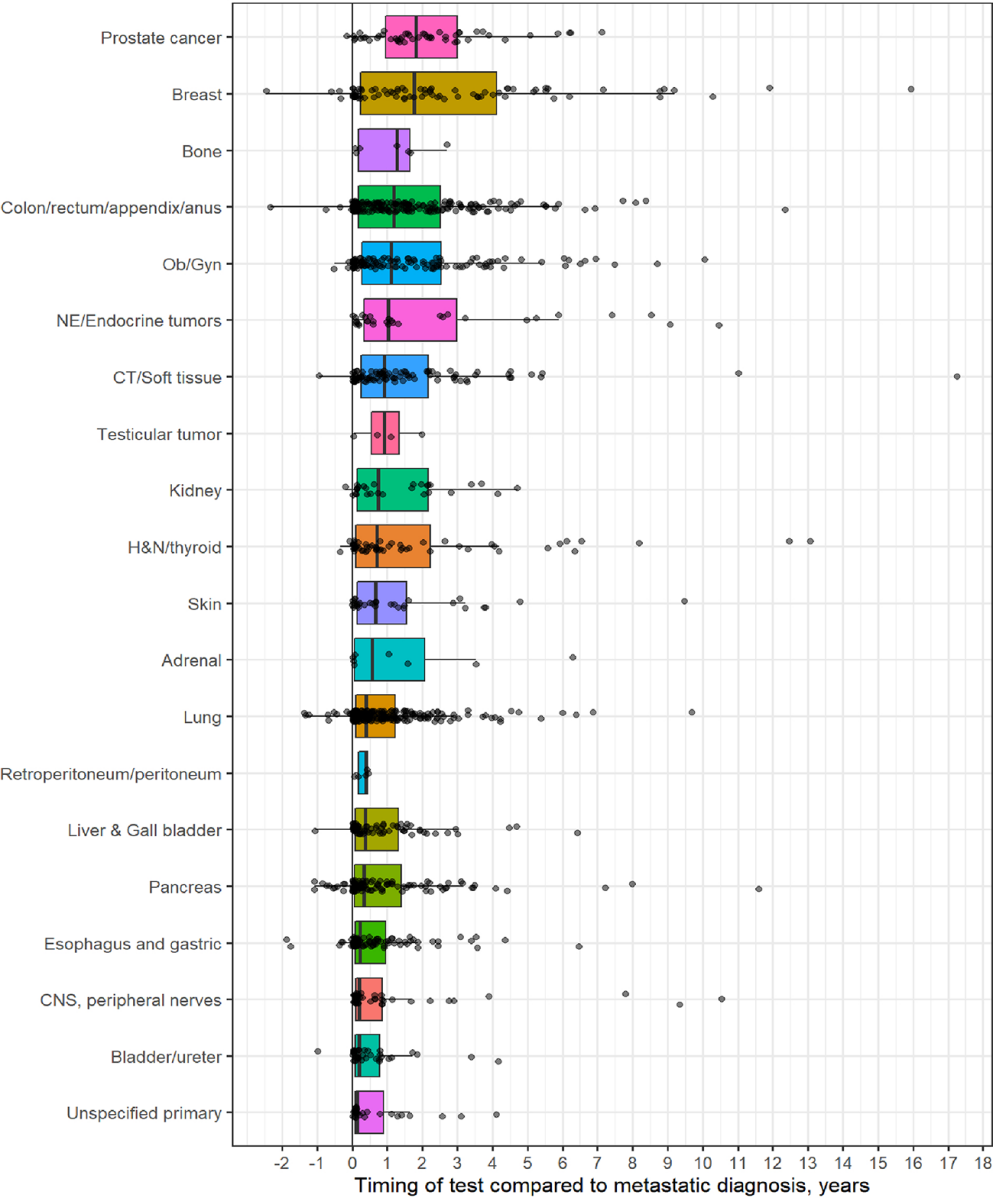
Distribution of comprehensive genomic profiling (CGP) test for each diagnostic category (Y-axis) to timing of CGP test (X- axis, time in years). On X-axis, 0 represent time of metastatic diagnosis and grey dots depicts timing of CGP test with respect to each diagnostic category.

**Table 1** outlines demographic distribution, disease categories tested and the interval between diagnosis and CGP and **Figure 1** depicts timing pattern of CGP for each diagnosis category.

**Table 1:** Characteristics and median time between metastatic diagnosis and CGP test of entire cohort who underwent comprehensive genomic profiling test after diagnosis of metastatic disease.

Characteristic	N	N = 1,358	Median test time, months	p-value
**Diagnosis category**	1,358			<0.001
CNS, peripheral nerves		42 (3.1%)	2.6 (-1.1, 6.4)	
H&N/thyroid		57 (4.2%)	8.5 (5.2, 12)	
Lung		254 (19%)	4.8 (3.3, 6.3)	
Breast		79 (5.8%)	21 (18, 24)	
Liver and Gall bladder		76 (5.6%)	4.6 (1.8, 7.3)	
Pancreas		106 (7.8%)	4.1 (1.7, 6.4)	
Esophagus and gastric		94 (6.9%)	2.6 (0.06, 5.1)	
Colon/rectum/appendix/anus		203 (15%)	14 (12, 16)	
Kidney		29 (2.1%)	8.9 (4.4, 13)	
Bladder/ureter		38 (2.8%)	2.4 (-1.6, 6.3)	
Adrenal		8 (0.6%)	10 (1.8, 19)	
Retroperitoneum/peritoneum		5 (0.4%)	4.7 (-6.1, 16)	
Ob/Gyn		121 (8.9%)	13 (11, 16)	
CT/Soft tissue		95 (7.0%)	11 (8.4, 13)	
Skin		31 (2.3%)	8.0 (3.6, 12)	
Bone		7 (0.5%)	15 (6.1, 25)	
Unknown primary		28 (2.1%)	1.7 (-2.9, 6.3)	
NE/Endocrine tumors		31 (2.3%)	12 (7.9, 17)	
Prostate cancer		50 (3.7%)	22 (19, 26)	
Testicular tumor		4 (0.3%)	11 (-1.2, 23)	
**Sex**	1,358			0.093
Female		710 (52%)	8.9 (7.3, 11)	
Male		648 (48%)	6.9 (5.2, 8.6)	
**Race, grouped**	1,358			0.8
Black or African American		186 (14%)	8.1 (5.0, 11)	
Others**^1^**		63 (4.6%)	9.6 (4.4, 15)	
White or Caucasian		1,109 (82%)	7.9 (6.6, 9.1)	
**Ethnicity**	1,356			0.4
Hispanic		36 (2.7%)	11 (3.9, 18)	
Non-Hispanic		1,320 (97%)	8.0 (6.8, 9.2)	
Unknown		2		

Lung and GI malignancies were prevalent in the entire cohort that underwent CGP. Caucasian and non-Hispanic comprised majority of the patient population in our study.

^1^Other racial groups included American Indian or Alaska Native (n=6), Asian (n=21), Native Hawaiian or Other Pacific Islander (n=1), Other (n=24), and Unknown (n=11).

CI, Confidence interval; CNS, Central Nervous system; H&N, Head and Neck cancers; Ob/Gyn, Obstetrics and gynecological cancers; CT, Connective tissues cancers; NE, Neuroendocrine tumors.

**Figure 2:**
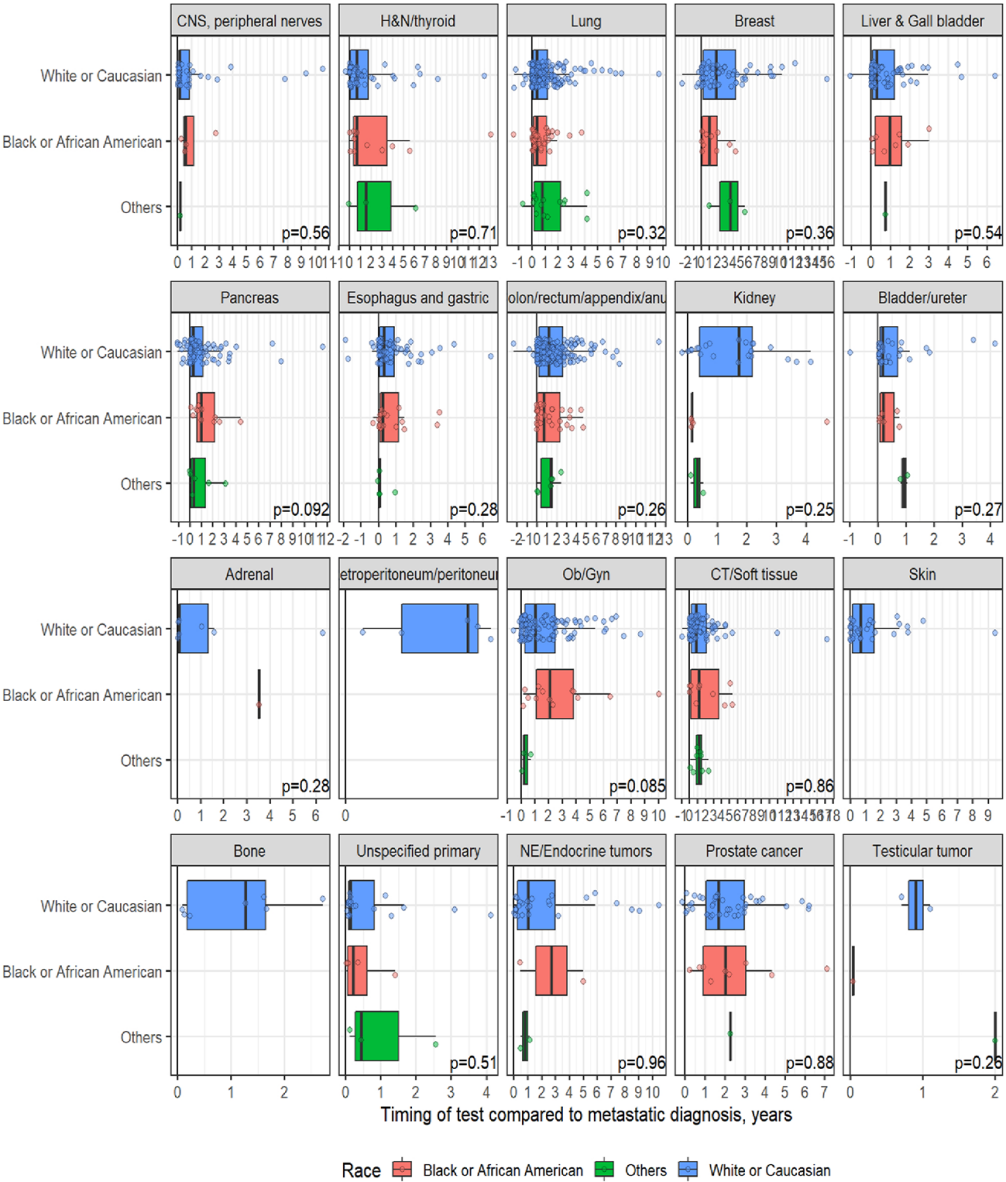
Effect of Race on timing of comprehensive genomic profiling (CGP) test after adjusting for diagnosis.

**Figure 3:**
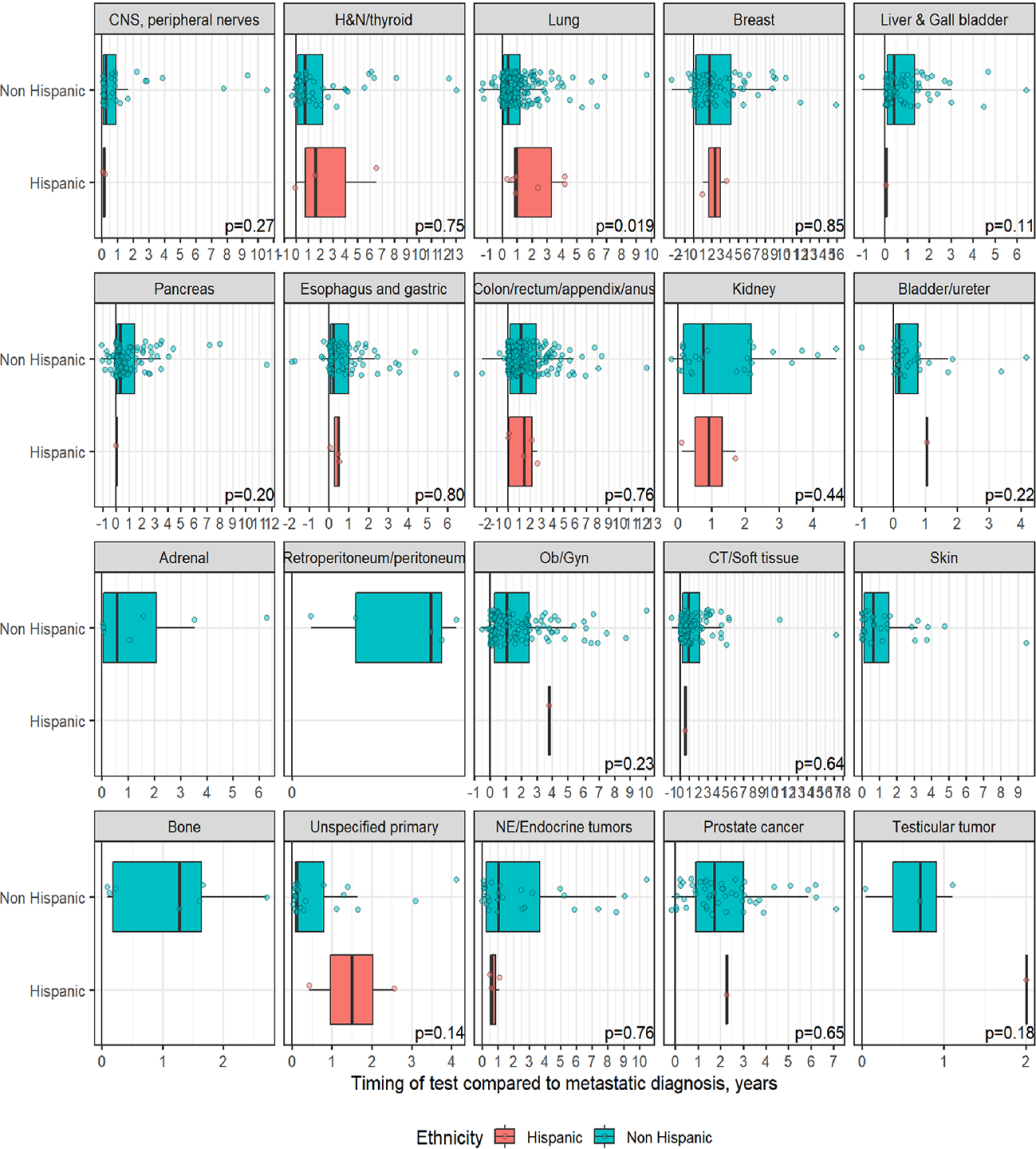
Effect of ethnicity on timing of comprehensive genomic profiling (CGP) test after adjusting for diagnosis. No significant difference in timing of CGP test observed except for lung cancer.

A total of 1,358 patients were identified for CGP - 710 (52%) were female; 1,109 (82%) were Caucasian, 186 (14%) were African American, and 36 (2.7%) patents were Hispanic (**Table 1**). Lung (254; 19%), lower GI tract - colorectal/anal/appendix (203; 15%), gynecologic (121; 8.9%), pancreatic (106; 7.8%) and connective tissue/soft tissue cancers (95; 7%) were the most common malignancies in the data set (**Table 1**). The median time to CGP after metastatic diagnoses was the shortest for Carcinoma of Unknown Primary (CUP) at 1.7 months and the longest for prostate cancer at 22 months. Several tumor types had a median time to CGP after metastatic diagnoses > 6 months. No significant racial differences were noted for time interval between metastatic diagnoses of cancers and CGP after adjusting for diagnosis (**Figure 2**). Overall, sex and ethnicity had no impact on time interval between metastatic diagnoses and CGP after adjusting for diagnosis with two exceptions – females with pancreatic cancer had a longer median time to CGP compared to males (p =0.025) and Hispanics with lung cancer had delayed CGP compared non-Hispanics (p=0.019) (**Figure 3**, **Supplementary Figure 1**).

In the overall model, we analyzed the effect of diagnosis category, race, and ethnicity on median time to CGP (**Supplementary table 1**). Compared to the Colon/rectum/appendix/anus, the diagnosis group with the largest number of patients, shorter differences in median (months) of -11, -8.5, -8.8, -8.3 were observed in patients diagnosed with CNS, lung, pancreas, and liver/gall bladder cancers, respectively, while longer median time differences (months) of 8 and 8.7 were observed for breast and prostate cancers, respectively.

**Figure 4:**
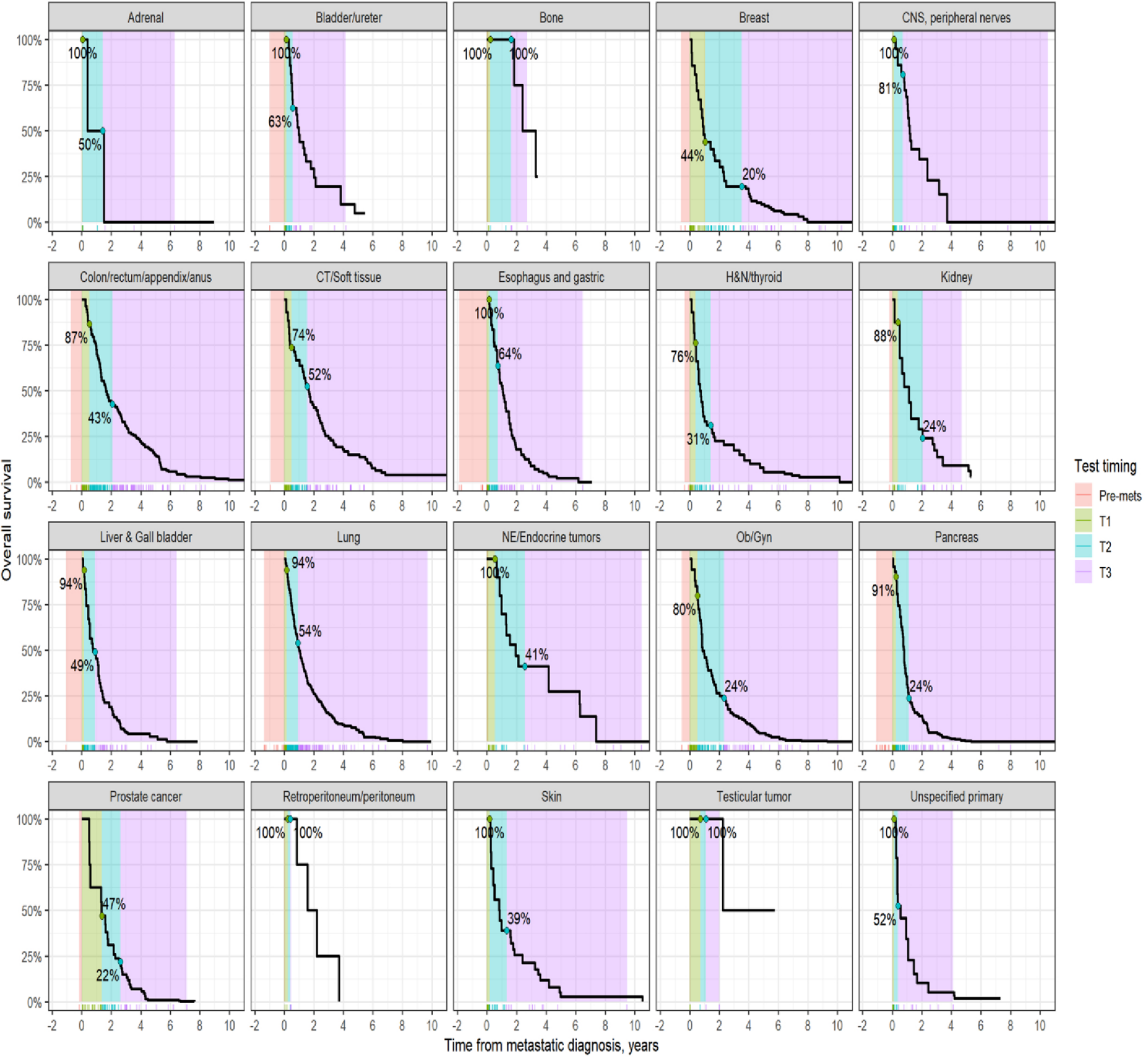
Kaplan-Meier estimates of overall survival by diagnosis. The shaded areas correspond to the diagnosis-specific groupings of time to CGP test, with the colored tick-marks at the bottom showing the actual CGP test times. The unshaded areas are outside the observed range of CGP test times. The two labeled points show the estimated survival at the time when 1/3^rd^ and 2/3^rd^ of post-met CGP tests have occurred.

**Figure 5:**
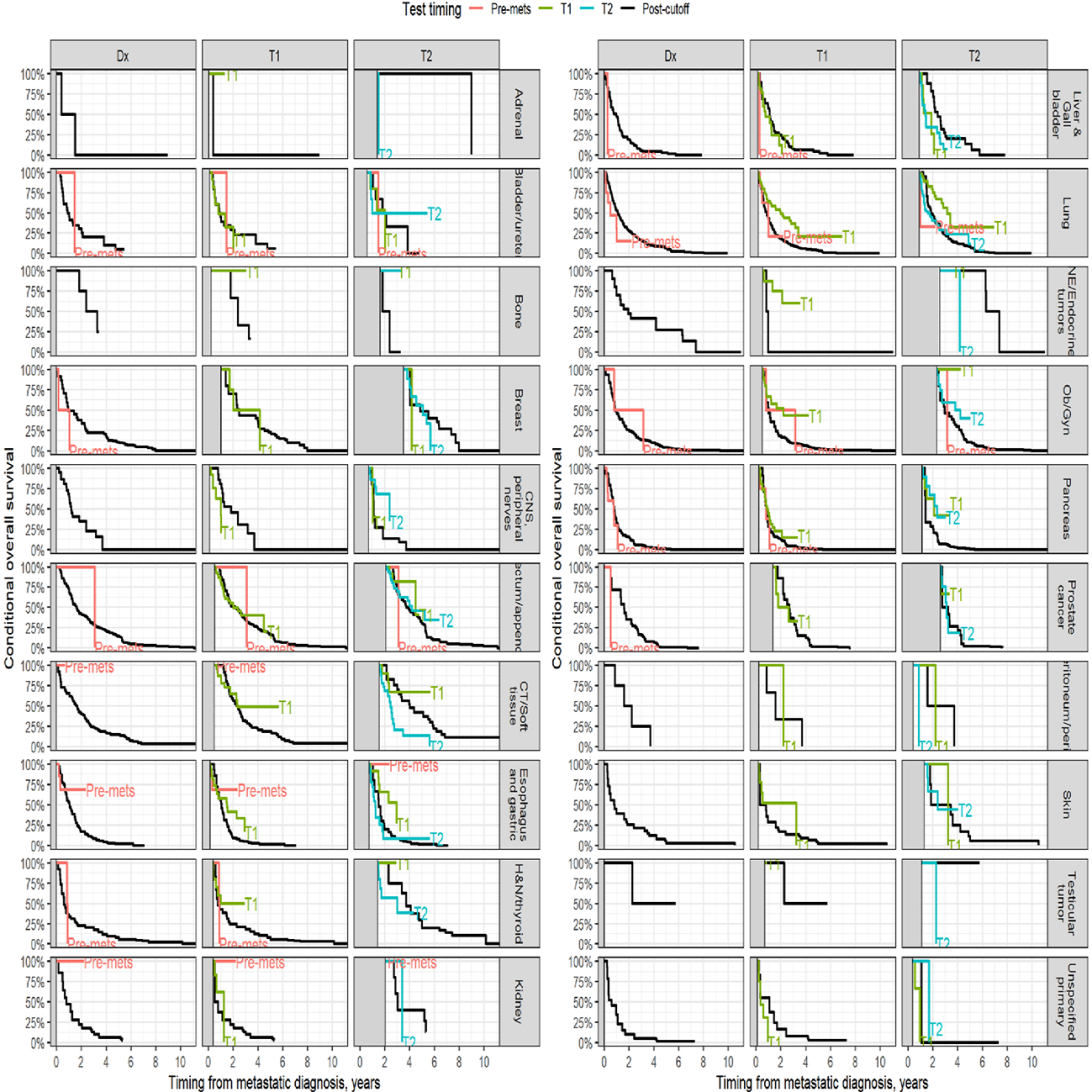
Kaplan-Meier estimates of conditional survival. For a visual comparison of the effect of the timing of the CGP test, conditional survival probabilities were computed. For each conditioning time (diagnosis, 1st and 2nd tertiles of the diagnostic category-specific distribution of test times), only patients alive without loss to follow up at the conditioning time were included.

**Figure 6:**
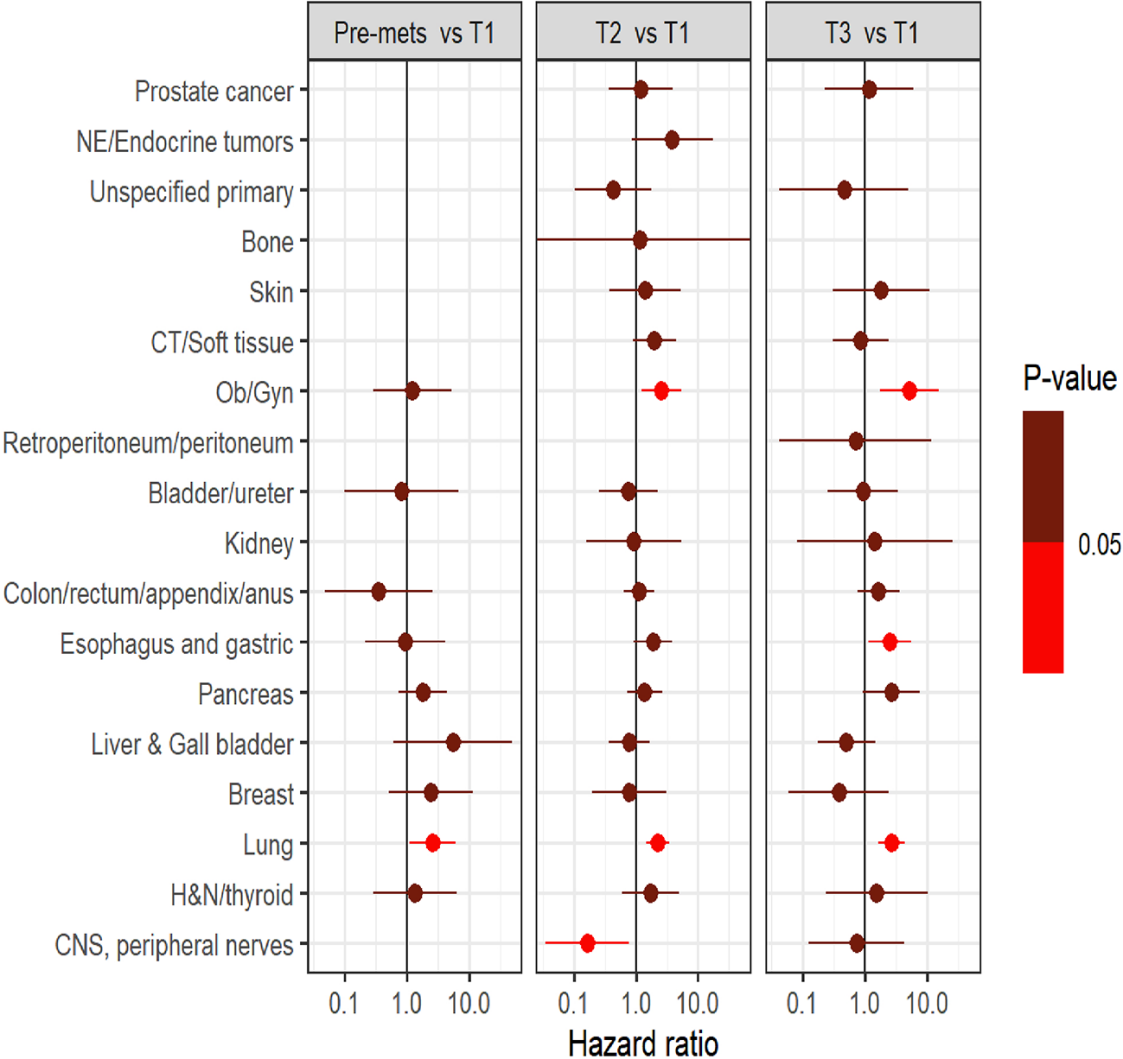
Comparison of survival outcome between groups (pre-mets, three tertiles namely T1, T2, and T3) within each diagnostic category.

Overall survival:

Survival analysis was performed on the entire data set except for 47 subjects with no follow-up data available after CGP. As described in the methods section, patients in each diagnostic category were divided into two broad groups- patients who had CGP performed before a formal metastatic diagnosis (pre-mets) and those who had CGP performed after metastatic diagnosis. Patients who had CGP performed after metastatic diagnosis were divided into diagnostic-category specific tertiles based on the time interval from metastatic diagnosis to CGP (T1- closest to diagnosis, T3 -furthest). **Figure 4** shows Kaplan-Meier survival by diagnosis with background coloring showing the tertiles of the test timing. In most of the diagnostic groups, the first third of the tests occur close to the time of the metastatic diagnosis, while the second third of tests happen by the median survival time. Breast and prostate cancer are notable exceptions, with fewer than a third of the tests occurring by the median survival time. Since the time of the CGP is not known at the time of metastatic diagnosis, patients cannot be directly grouped by the CGP time tertiles. **Figure 5** depicts Kaplan-Meier estimates of OS conditional on being alive at consecutive tertiles of the testing-time distribution. In each column patients are grouped according to the timing of their test, with all patients who have not had a test yet combined into a “post-cutoff” group with their estimate survival curve drawn in black. The plots show substantial variability, but earlier testing times, especially the first tertile T1, tend to have better survival then the post-cutoff group. In a formal evaluation, Cox proportional hazards regression model stratified by diagnostic groups (**Table 2**) demonstrated that early testing had an impact on survival with patients who had CGP performed in the second or third tertile after metastatic diagnosis having HRs of 1.53 (95% CI 1.25, 1.87; p < 0.001) and 1.86 (95% CI 1.42, 2.42; p<0.001) respectively, compared to patients in the first tertile. Patients with CGP before the metastatic diagnosis had a slightly elevated hazard of death that did not reach statistical significance (HR=1.35, 95% CI 0.88, 2.07; p=0.2). Subset analyses by diagnostic group showed significant effects within the lung, gastro-esophageal and gynecologic malignancies (**Supplementary table 2**, **Figure 6**). Patients with lung cancer who had CGP performed prior to metastatic diagnosis as well as the second or third tertile after metastatic diagnosis had HRs of 2.62 (95% CI 1.10, 6.24; p = 0.029), 2.25 (95% CI 1.48, 3.41; p<0.001) and 2.67 (95% CI 1.61, 4.42; p<0.001) respectively, compared to patients in the first tertile. Patients with gastro-esophageal malignancies who had CGP performed in the third tertile after metastatic diagnosis had HR of 2.49 (95% CI 1.13, 5.49; p = 0.024) compared to patients in the first tertile. Patients with gynecologic malignancies who had CGP performed in the second or third tertile after metastatic diagnosis had HRs of 2.58 (95% CI 1.23, 5.41; p = 0.012) and 5.19 (95% CI 1.74, 5.19; p = 0.003) respectively, compared to patients in the first tertile. Additionally, among patients with CNS malignancies, patients who had CGP performed in the second tertile, had significantly lower mortality compared to the first tertile (HR = 0.17, 95% CI 0.04, 0.77; p=0.022).

**Table 2:** Cox proportional hazards regression model stratified by diagnostic groups.

Characteristic	N	Event N	HR	95% CI	p-value
Test timing					
T1	21	222	—	—	
Pre-mets	42	25	1.35	0.88, 2.07	0.2
T2	417	273	1.53	1.25, 1.87	<0.001
T3	431	298	1.86	1.42, 2.42	<0.001

Overall survival benefit was noted in patients who had CGP testing performed in the second or third tertile after metastatic diagnosis.

^1^ HR, Hazard Ratio; CI, Confidence Interval.

## Discussion

In this study significant differences were noted among various cancer types in the interval between metastatic diagnosis and CGP – Unknown primary had the shortest interval while breast and prostate cancers had the longest interval. We found no difference in the interval between metastatic diagnosis and CGP based on race, ethnicity, and sex with the following exceptions - Hispanics with lung cancer and females with pancreatic cancer were noted to have delayed CGP compared to non-Hispanics and males, respectively. Overall, this study demonstrated the variability in institutional practice pertaining to CGP among various cancer types, and more importantly highlighted the need for consensus guidelines to ensure optimal selection, and implementation of cancer precision medicine assays.

Improved survival was observed in patients with lung, gastroesophageal and gynecologic cancers when CGP was performed earlier in their treatment program. However, interpretation of OS data from this study merits caution as CGP was performed for different cancer types at different time points after their metastatic diagnoses. Nevertheless, early CGP testing in patients with advanced malignancies, irrespective of race and ethnicity, could potentially impact overall clinical outcomes due to their prognostic and predictive impact.

In a real-world setting, Singh et al. demonstrated identification of potentially actionable genomic alterations in all cancer types using CGP; however, only 10% of patients experienced a change in cancer therapy based on the identification of actionable genomic alterations, notably, in lung cancer (8). Another retrospective study noted the low proportion of patients deriving therapeutic benefit from CGP; however, treatment was changed in 13.8% (20/145) patients with metastatic disease *vs*. 4% (1/25) without metastatic disease (p=0.09) (9). Authors reported changes in cancer therapies – on and off-label as well as clinical trial participation. The value of CGP in identifying genomic alterations that lead to participation in biomarker enriched clinical trials have been reported in other series as well (10, 11).

The evidence in support of early CGP is still evolving and there is significant variability in the interval between diagnosis to CGP among various metastatic solid tumors. This is at least in part, due to the lack of viable targeted therapies for most genomic alterations detected using high throughput somatic CGP panels (12). Further, clonal diversity, spatiotemporal heterogeneity, various cellular as well as extracellular signaling pathways that contribute to therapeutic resistance, and complex tumor microenvironments often limit the durability of targeted therapies, tempering the enthusiasm for CGP in at least some practitioners (12).

There are significant variabilities in institutional practice as it pertains to the platform used for CGP, both regionally, and nationally. This variability is present between disease ontologies/histologic subtypes and sometime, even among providers within the same disease-oriented team. As CGP became an integral part of cancer care, we recognized the lack of a systematic process as well as organizational structure to determine optimal selection, and utilization of precision medicine assays within our matric cancer center.

**Figure 7:**
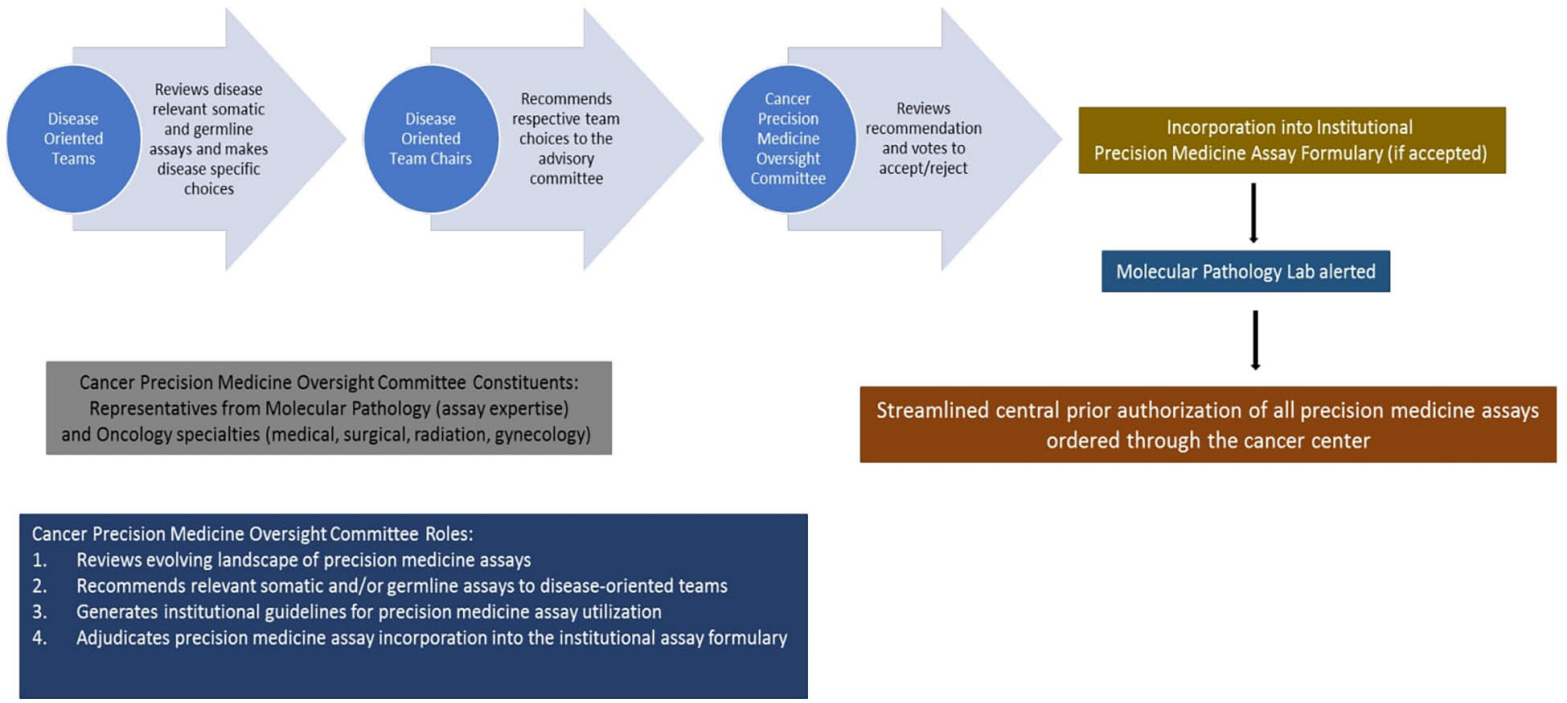
Proposed framework for selection, utilization, and implementation of cancer precision medicine assays based on current workflow at the Medical College of Wisconsin Cancer Center.

Therefore, we formulated an institutional Cancer Precision Medicine Oversight Committee (CPMOC) comprised of scientific and clinical experts responsible for systematically assessing the Precision Medicine Assays (PMA) available for routine clinical use and thus maintaining a robust PMA portfolio to support patient care. **Figure 7** depicts this algorithm with different steps involved. The CPMOC has multidisciplinary clinical representation and the requisite precision medicine expertise to effectively integrate precision medicine assays into clinical decision making, thus ensuring state of the art patient care. This committee also provides a systematic assessment of existing and evolving cancer-specific assays in the precision medicine landscape and makes recommendations to maintain a scientifically robust and operationally efficient PMA portfolio. Additionally, this committee provides input to clinical informatics partners to streamline effective clinical workflows, support assay ordering, resulting, and disposition of actionable results from PMAs.

## Limitations

There are several limitations to our single institution study. First, the retrospective nature of the study comes with the expected, inherent confounders. Second, we divided patients based on functional organ systems rather than histologic subtypes. Since solid tumors are biologically heterogeneous despite similar histo-morphological features, we acknowledge that this is a weakness. Despite having a relatively large data set, we felt that narrowing the analysis to each histologic subtype would significantly compromise our ability to draw any meaningful conclusions. Third, we acknowledge the limitations imposed by the lack of a robust multivariate analysis – a problem inherent to this type of retrospective study. Fourth, while many commercial CGP panels are available for patients with advanced malignancies, our effort analyzes the impact of ‘foundation one’ - the most commonly used CGP panel at our institution – in routine clinical care. While this allows for homogeneity in terms of prior authorization processes (as it pertains to insurance coverage), we acknowledge that this data cannot be extrapolated to ascertain the impact of other commercially available CGPs. Fifth, we did not consider age to evaluate any health disparities for CGP testing since our primary goal as to ascertain the impact of ‘timing of CGP testing’ irrespective of patient age. We also acknowledge that the sample size comprises mainly Caucasian and Non-Hispanic populations, which makes it quite challenging to apply results from this study to different demographics in other places or countries.

Finally, pre-selection bias cannot be excluded as clinicians have different threshold to order CGP testing for metastatic disease. It is quite possible that patients with multiple standards of care treatment options and longer natural history (such as metastatic breast and prostate cancers) had CGP performed later in their disease course than patient’s aggressive malignancies with limited treatment options. Further, provider tendencies to order CGP likely increased over time as well. Despite the limitations, this study offers valuable insight into the variabilities in CGP utilization patterns in a large matrix cancer center, emphasizing the need for organizational structure and consensus guidelines to optimize selection and utilization of PMAs.

## Conclusions

Since cure is limited to a small subset of patients with metastatic solid tumors, CGP is an avenue for maximizing therapeutic options, leveraging precision oncology trials and off-label therapies based on consensus treatment recommendations derived from institutional molecular tumor boards. There are significant differences in awareness among oncology practitioners regarding analysis and interpretation of CGP data. It is necessary to initiate Continuing Medication Education in this area to maximize awareness of this rapidly evolving field and to institute ‘best practice’. Further, there is an urgent need to generate consensus guidelines on patient selection for CGP and optimal timing for testing. Quality initiatives that capture these metrics need to be incorporated into routine clinical practice. Further, detailed cost-benefit analysis of routine CGP in patients with metastatic solid tumors will help the oncology community determine the true value of such testing.

## Data availability statement

The raw data supporting the conclusions of this article will be made available by the authors, without undue reservation.

## Ethics statement

The studies involving human participants were reviewed and approved by Medical College of Wisconsin. Written informed consent for participation was not required for this study in accordance with the national legislation and the institutional requirements.

## Author contributions

BT and GA: Designed research, Data collection, and Manuscript writing. AS: Data Analysis, critically reviewed and approved the manuscript. MK, DK, MM, SM, SD, JT, and JaT: Critically reviewed and approved the manuscript. BG: Designed research and Manuscript writing. All authors contributed to the article and approved the submitted version.
