# Supplementary material for: Comprehensive genomic profiling: Does timing matter?
Source: Front Oncol. 2023 Feb 14;13:1025367. doi: 10.3389/fonc.2023.1025367 (PMC9971445; doi:10.3389/fonc.2023.1025367)
Supplement: Supplementary file 1 [file DataSheet_1.docx]

**
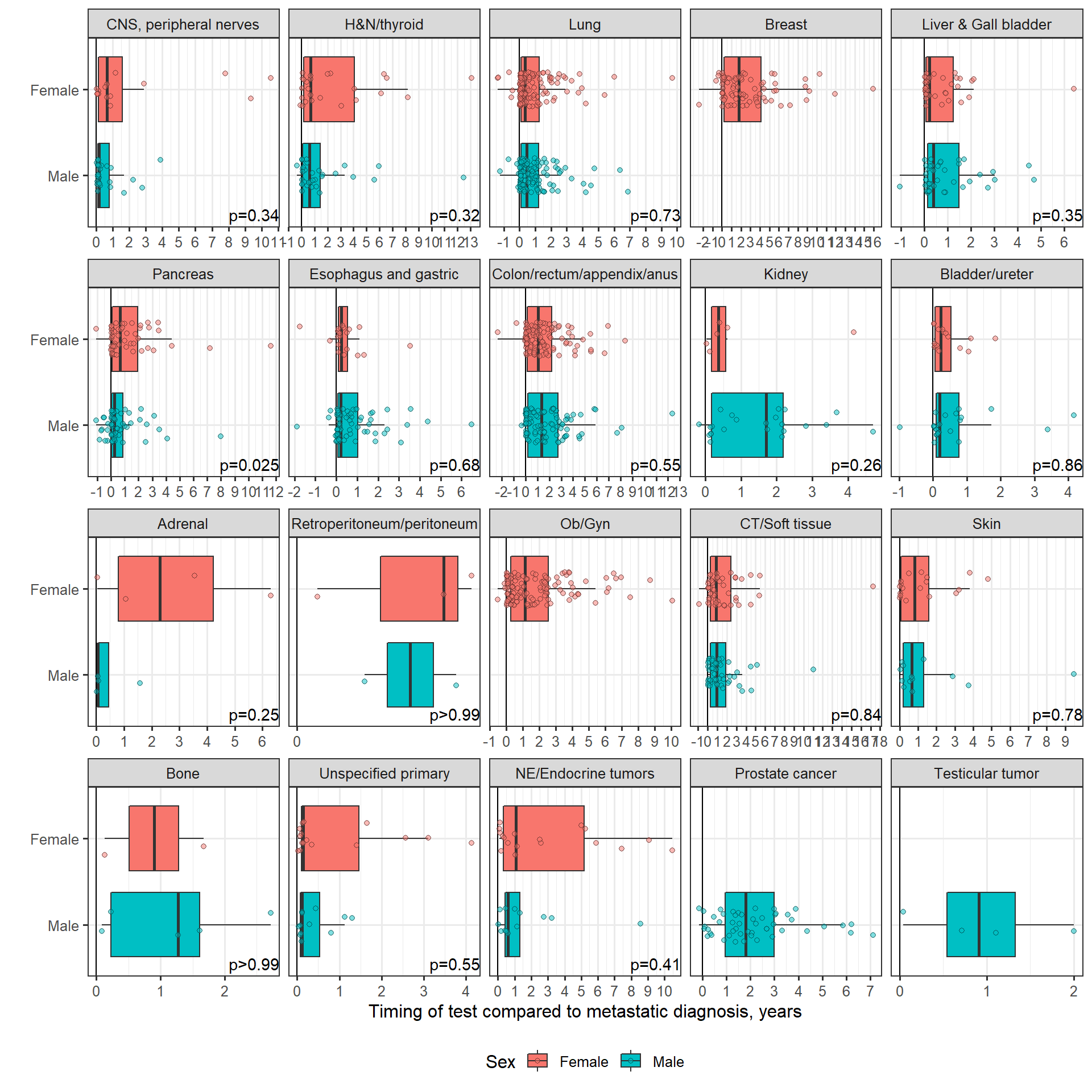
**

**Supplementary Figure 1:** Effect of gender on timing of comprehensive genomic profiling (CGP) test performed after adjusting for diagnosis. Females with pancreatic cancer showed significant difference in timing of CGP test, no significant difference in other diagnostic category.

CNS: Central Nervous system, H&N: Head and Neck cancers, Ob/Gyn: Obstetrics and gynecological cancers, CT: Connective tissues cancers, NE: Neuroendocrine tumors

| **Characteristic** | **Difference of Medians** | **95% CI*^1^*** | **p-value** |
| --- | --- | --- | --- |
| (Intercept) | 13 | 11, 15 | <0.001 |
| **Diagnosis** |  |  | <0.001 |
| Colon/rectum/appendix/anus | — | — |  |
| CNS, peripheral nerves | -11 | -14, -7.3 | <0.001 |
| H&N/thyroid | -4.8 | -7.8, -1.7 | 0.002 |
| Lung | -8.5 | -10, -6.5 | <0.001 |
| Breast | 8.0 | 5.3, 11 | <0.001 |
| Liver & Gall bladder | -8.3 | -11, -5.6 | <0.001 |
| Pancreas | -8.8 | -11, -6.3 | <0.001 |
| Esophagus and gastric | -11 | -13, -8.3 | <0.001 |
| Kidney | -3.8 | -7.9, 0.18 | 0.061 |
| Bladder/ureter | -11 | -14, -7.2 | <0.001 |
| Adrenal | -3.3 | -11, 4.0 | 0.4 |
| Retroperitoneum/peritoneum | -8.3 | -17, 0.86 | 0.076 |
| Ob/Gyn | -0.59 | -3.0, 1.8 | 0.6 |
| CT/Soft tissue | -2.3 | -4.8, 0.25 | 0.078 |
| Skin | -4.8 | -8.7, -0.90 | 0.016 |
| Bone | 2.5 | -5.2, 10 | 0.5 |
| Unspecified primary | -11 | -15, -7.2 | <0.001 |
| NE/Endocrine tumors | -1.1 | -5.0, 2.9 | 0.6 |
| Prostate cancer | 8.7 | 5.5, 12 | <0.001 |
| Testicular tumor | -2.0 | -12, 8.3 | 0.7 |
| **Race, grouped** |  |  | 0.065 |
| White or Caucasian | — | — |  |
| Black or African American | 1.9 | 0.31, 3.5 | 0.020 |
| Others | 0.45 | -2.6, 3.5 | 0.8 |
| **Sex** |  |  | 0.7 |
| Female | — | — |  |
| Male | -0.23 | -1.5, 1.0 | 0.7 |
| **Ethnicity** |  |  | 0.052 |
| Non-Hispanic | — | — |  |
| Hispanic | 4.0 | -0.03, 8.0 | 0.052 |
| ^1^ CI = Confidence Interval | | | |

**Supplementary table 1: Overall model analyzing effect of diagnostic category, race, and ethnicity on median time to CGP**

| **Characteristic** | **N** | **Event N** | **HR** | **95% CI** | **p-value** |
| --- | --- | --- | --- | --- | --- |
| CNS, peripheral nerves |  |  |  |  |  |
| T1 | 14 | 6 | — | — |  |
| T2 | 14 | 5 | 0.17 | 0.04, 0.77 | 0.022 |
| T3 | 14 | 10 | 0.73 | 0.13, 4.20 | 0.7 |
| H&N/thyroid |  |  |  |  |  |
| T1 | 18 | 9 | — | — |  |
| Pre-mets | 2 | 2 | 1.35 | 0.29, 6.34 | 0.7 |
| T2 | 18 | 11 | 1.70 | 0.59, 4.88 | 0.3 |
| T3 | 19 | 11 | 1.54 | 0.23, 10.2 | 0.7 |
| Lung |  |  |  |  |  |
| T1 | 80 | 41 | — | — |  |
| Pre-mets | 9 | 6 | 2.62 | 1.10, 6.24 | 0.029 |
| T2 | 80 | 60 | 2.25 | 1.48, 3.41 | <0.001 |
| T3 | 80 | 59 | 2.67 | 1.61, 4.42 | <0.001 |
| Breast |  |  |  |  |  |
| T1 | 25 | 12 | — | — |  |
| Pre-mets | 2 | 2 | 2.42 | 0.51, 11.4 | 0.3 |
| T2 | 24 | 13 | 0.78 | 0.20, 3.08 | 0.7 |
| T3 | 25 | 13 | 0.38 | 0.06, 2.39 | 0.3 |
| Liver & Gall bladder |  |  |  |  |  |
| T1 | 23 | 16 | — | — |  |
| Pre-mets | 1 | 1 | 5.51 | 0.61, 49.6 | 0.13 |
| T2 | 25 | 20 | 0.78 | 0.37, 1.65 | 0.5 |
| T3 | 24 | 17 | 0.50 | 0.17, 1.46 | 0.2 |
| Pancreas |  |  |  |  |  |
| T1 | 29 | 21 | — | — |  |
| Pre-mets | 11 | 7 | 1.80 | 0.74, 4.35 | 0.2 |
| T2 | 29 | 21 | 1.39 | 0.73, 2.61 | 0.3 |
| T3 | 30 | 26 | 2.68 | 0.94, 7.66 | 0.065 |
| Esophagus and gastric |  |  |  |  |  |
| T1 | 27 | 15 | — | — |  |
| Pre-mets | 7 | 2 | 0.93 | 0.21, 4.08 | >0.9 |
| T2 | 27 | 19 | 1.88 | 0.93, 3.79 | 0.079 |
| T3 | 27 | 22 | 2.49 | 1.13, 5.49 | 0.024 |
| Colon/rectum/appendix/anus |  |  |  |  |  |
| T1 | 65 | 33 | — | — |  |
| Pre-mets | 3 | 1 | 0.35 | 0.05, 2.58 | 0.3 |
| T2 | 64 | 34 | 1.11 | 0.63, 1.95 | 0.7 |
| T3 | 65 | 43 | 1.65 | 0.76, 3.58 | 0.2 |
| Kidney |  |  |  |  |  |
| T1 | 9 | 4 | — | — |  |
| Pre-mets | 1 | 0 | 0.00 | 0.00, Inf | >0.9 |
| T2 | 9 | 6 | 0.93 | 0.16, 5.47 | >0.9 |
| T3 | 10 | 5 | 1.43 | 0.08, 25.5 | 0.8 |
| Bladder/ureter |  |  |  |  |  |
| T1 | 11 | 7 | — | — |  |
| Pre-mets | 1 | 1 | 0.83 | 0.10, 6.87 | 0.9 |
| T2 | 13 | 7 | 0.75 | 0.25, 2.21 | 0.6 |
| T3 | 12 | 6 | 0.93 | 0.25, 3.39 | >0.9 |
| Adrenal |  |  |  |  |  |
| T1 | 3 | 0 | — | — |  |
| T2 | 2 | 2 | Inf | 0.00, Inf | >0.9 |
| T3 | 3 | 1 | — | — |  |
| Retroperitoneum/peritoneum |  |  |  |  |  |
| T1 | 2 | 1 | — | — |  |
| T2 | 1 | 1 | Inf | 0.00, Inf | >0.9 |
| T3 | 2 | 2 | 0.71 | 0.04, 11.8 | 0.8 |
| Ob/Gyn |  |  |  |  |  |
| T1 | 38 | 20 | — | — |  |
| Pre-mets | 3 | 2 | 1.22 | 0.28, 5.28 | 0.8 |
| T2 | 36 | 24 | 2.58 | 1.23, 5.41 | 0.012 |
| T3 | 39 | 30 | 5.19 | 1.74, 15.5 | 0.003 |
| CT/Soft tissue |  |  |  |  |  |
| T1 | 30 | 15 | — | — |  |
| Pre-mets | 1 | 0 | 0.00 | 0.00, Inf | >0.9 |
| T2 | 30 | 22 | 1.98 | 0.89, 4.40 | 0.094 |
| T3 | 31 | 18 | 0.85 | 0.30, 2.40 | 0.8 |
| Skin |  |  |  |  |  |
| T1 | 10 | 5 | — | — |  |
| T2 | 10 | 7 | 1.41 | 0.38, 5.23 | 0.6 |
| T3 | 11 | 8 | 1.82 | 0.30, 10.9 | 0.5 |
| Bone |  |  |  |  |  |
| T1 | 2 | 0 | — | — |  |
| T2 | 2 | 0 | 1.16 | 0.00, Inf | >0.9 |
| T3 | 3 | 3 | Inf | 0.00, Inf | >0.9 |
| Unspecified primary |  |  |  |  |  |
| T1 | 8 | 6 | — | — |  |
| T2 | 8 | 4 | 0.43 | 0.10, 1.80 | 0.2 |
| T3 | 8 | 5 | 0.46 | 0.04, 5.06 | 0.5 |
| NE/Endocrine tumors |  |  |  |  |  |
| T1 | 10 | 3 | — | — |  |
| T2 | 9 | 6 | 3.84 | 0.86, 17.1 | 0.078 |
| T3 | 10 | 6 | 0.00 | 0.00, Inf | >0.9 |
| Prostate cancer |  |  |  |  |  |
| T1 | 16 | 8 | — | — |  |
| Pre-mets | 1 | 1 | Inf | 0.00, Inf | >0.9 |
| T2 | 15 | 10 | 1.20 | 0.36, 3.96 | 0.8 |
| T3 | 16 | 13 | 1.17 | 0.23, 6.05 | 0.9 |
| Testicular tumor |  |  |  |  |  |
| T1 | 1 | 0 | — | — |  |
| T2 | 1 | 1 | Inf | 0.00, Inf | >0.9 |
| T3 | 2 | 0 | — | — |  |

**Supplementary Table 2:** Effect of timing of CGP test on survival based on diagnostic category**.** Cox proportional hazards regression with test-time groups as a time-dependent covariate was fitted to the overall dataset and within each diagnostic category.

HR: Hazard Ratio, CI: Confidence Interval, CNS: Central Nervous system, H&N: Head and Neck cancers, Ob/Gyn: Obstetrics and gynecological cancers, CT: Connective tissues cancers, NE: Neuroendocrine tumors
